# Portal Hypertensive Polyps as Gastroscopic Finding in Liver Cirrhosis

**DOI:** 10.1155/2020/9058909

**Published:** 2020-02-26

**Authors:** Firdevs Topal, Sabiye Akbulut, Cengiz Karahanlı, Süleyman Günay, Elif Sarıtaş Yüksel, Fatih Esad Topal

**Affiliations:** ^1^Department of Gastroenterology, Katip Celebi University Ataturk Training and Research Hospital, 35360 Izmir, Turkey; ^2^Department of Gastroenterology, University of Health Sciences, Kartal Koşuyolu High Specialty Training and Research Hospital, 34846 Istanbul, Turkey; ^3^Department of Emergency, Katip Celebi University Ataturk Training and Research Hospital, 35360 Izmir, Turkey

## Abstract

**Background:**

Portal hypertensive polyps in patients with portal hypertension are described.

**Aims:**

The most significant and serious complication in liver cirrhosis proves to be portal hypertension. Polypoid lesions, which can be seen in the stomach as endoscopic finding in patients with portal hypertension, have not quite been defined in the literature. The aim of this study, therefore, was to define polypoid lesion formation due to portal hypertension in the upper gastrointestinal system in patients with portal hypertension. *Study Design*. Cross-sectional study.

**Methods:**

The study covered a group of patients with liver cirrhosis and a healthy control group that did not have portal hypertension. All individuals covered by the study received upper GI endoscopy, while the endoscopic features and pathological characteristics of the identified polypoid lesions were defined. Standard histological criteria were used in polyp diagnosis.

**Results:**

A total of 400 individuals were included in the study. Upper GI endoscopy was performed for 200 patients with liver cirrhosis and another 200 healthy individuals with no portal hypertension in the control group. When the cases were gastroscopically assessed with regard to polypoid lesion presence, it was seen that a total of 87 (21.8%) individuals had polyps. While 67 (33.5%) cirrhotic patients were identified to have polyps, 20 (10%) individuals in the healthy control group had polyps. When the results of those with liver cirrhosis who received esophageal variceal endoscopic band ligation (EVL) and who did not were compared, it was observed that a higher number of individuals in the group with EVL had polypoid lesions. When the patient and control groups were compared as to Helicobacter pylori presence, the results showed that it was slightly higher in the dyspepsia group but the difference was not statistically significant (*p* > 0.05).

**Conclusion:**

Portal hypertension-associated polypoid lesions are common in advanced liver cirrhosis cases. The pathological analyses of these polyps pointed out that they were all benign and no malignant cases were detected. It was argued that these polypoid lesions, referred to as portal hypertensive polyps, were associated with elevated angiogenesis in the gastric mucosa.

## 1. Introduction

Esophageal varices, fundal varices, and portal hypertensive gastropathy are frequently observed and well-defined findings with a well-known mechanism during upper GI endoscopy in patients with liver cirrhosis. The most common mucosal damage seen in patients with liver cirrhosis is portal hypertensive gastropathy (PHG). Although the exact pathogenesis of PHG is unclear, it is held to mainly involve local or generalized alterations in vascular hemodynamics [1]. In addition to such findings, polypoid lesions in the stomach have rarely been defined as endoscopic findings in patients with liver cirrhosis [2–4]. The results of certain studies have revealed that such polypoid formations were characterized histologically by the dilatation in the mucosal and submucosal vessels of the stomach [3, 4].

The aim of this study was to investigate the frequency of gastric polypoid lesions in patients diagnosed with liver cirrhosis through upper GI endoscopy, to compare these results with those of the healthy control group, and to present histological findings.

## 2. Materials and Methods

Gastric polypoid lesions detected through upper GI endoscopy in the patient group with liver cirrhosis as well as the healthy control group were evaluated within the scope of this study. The results of cirrhotic patients who received esophageal variceal endoscopic band ligation (EVL) and those who did not were further comparatively evaluated. The causes of liver cirrhosis were retrospectively identified in patients with liver cirrhosis. Diagnostic criteria for portal hypertension were set as the presence of esophageal and/or fundal varices, ascites, splenomegaly, and portal hypertensive gastropathy while the presence of one or two of these was singled out as the diagnostic criterion for portal hypertension. Biopsy samples were collected from all polyps detected through gastroscopy and were transferred to the pathology department for histopathological analyses. The diagnosis of polyps was established according to standard histological criteria. The presence of Helicobacter pylori was ascertained through hematoxylin and eosin and Giemsa staining within the scope of the histopathological analyses of these polyp samples. This study was approved by the institutional committee of ethics.

### 2.1. Study Population

200 patients diagnosed with liver cirrhosis (etiological factors are outlined in Table 1) comprised the study group while 200 others who had presented due to dyspepsia with no signs of hypertension and for whom gastroscopy procedures had been planned comprised the healthy control group. Gastroscopy was performed for both groups, i.e., for a total of 400 individuals. 118 (59%) cirrhotic male patients and 82 (41%) cirrhotic female patients, making a total of 200 patients, comprised the study group whose mean age was 62.13 ± 12.29 years (age range: 27-90). The 200-patient strong control group was made up of 89 (44.5%) male patients and 111 (55.5%) female patients whose mean age was 52.35 ± 16.01 years (age range: 18-87). There was no statistically significant difference between the two groups with regard to either age or sex (*p* > 0.05) Table 2 presents the summary of both groups' demographic data.

Biopsy samples were collected from the antrum and corpus during the upper GI endoscopy along with biopsy samples from the identified polyps. Experienced pathologists performed the pathological analyses of these biopsy samples according to histological criteria at the pathology department.

### 2.2. Statistical Analysis

The data collected within the scope of the study were analyzed by the IBM SPSS Statistics 25.0 (IBM Corp., Armonk, New York, US) software. Since it was a retrospective study, post hoc analysis was used instead of sample size calculation. Descriptive statistical data were presented as unit number (*n*), percentage (%), and mean ± standard deviation (*x* ± sd). The Shapiro-Wilk normality test and Q-Q graphics were used to assess the normal distribution of numeric variables. Between-group comparisons were conducted by using the independent two-sample *t*-test for normally distributed variables. The chi-square exact test was used to assess the relationship among the categorical variables. Intragroup comparisons were carried out by the two-proportion *z*-test with Bonferroni correction in cases where the results of the chi-square test were found to be significant. The backward elimination method of the binary logistic regression analysis was utilized to identify the factors affecting polypoid formation. *p* < 0.05 value was set as statistically significant.

## 3. Results

The post hoc analysis revealed a 100% post hoc power with a type I error as 0.05. When the etiologies causing cirrhosis were retrospectively analyzed in the cirrhotic group, it was observed that 63 (31.5%) out of a total of 200 patients had chronic hepatitis B-associated liver cirrhosis, 35 (17.5%) had cryptogenic cirrhosis, and 11 (5.5%) had chronic hepatitis C-associated liver cirrhosis. As is revealed by the results of our study, chronic hepatitis B-associated liver cirrhosis was in the lead.

Upper GI endoscopy was performed for a total of 400 individuals making up the study and control groups (Table 3). The polypoid lesions, identified in the corpus and antrum through gastroscopy for the cirrhotic and noncirrhotic groups, were compared. 87 (21.8%) persons were detected to have polypoid lesions as revealed by the results of a total of 400 gastroscopy procedures. 67 (33.5%) out of 200 cirrhotic patients had polypoid lesions, while 20 (10%) out of 200 patients in the control group had polypoid lesions. When a statistical analysis was conducted between the two groups as per the presence of polypoid lesions detected within the scope of gastroscopic assessment, it was seen that the number of persons with polypoid lesions in the cirrhotic group was higher than that in the control group with a statistically significant difference (*p* value < 0.001).

It was observed that the polyps were localized more in the antrum and prepyloric antrum of the stomach. The number of polyps per patient in the cirrhotic group varied between 4 and 11, while the size of their polyps ranged between 2 and 6 mm. The minimum number of polyps per person in the control group was 1, while the maximum number was 4. The sizes of the polyps in the control group were larger, ranging between 4 and 16 mm. These polyps were generally localized in the gastric antrum and corpus; they were single or multiple and sessile or pedunculated.

The biopsy samples collected from the antrum and corpus during the upper GI endoscopy were analyzed pathologically for Helicobacter pylori (H. pylori). H. pylori was detected less in the cirrhotic group, while it was observed slightly more in the dyspepsia group, but there was no statistically significant difference between the two groups (*p* < 0.09) Table 4.

When the results of those with liver cirrhosis who received esophageal variceal endoscopic band ligation (EVL) and who did not were compared, it was observed that a higher number of individuals in the group with EVL had polypoid lesions and they statistically faced 2.891 times more the risk of contracting polypoid formations.

## 4. Discussion

Portal hypertensive gastropathy (PHG) is a well-defined endoscopic finding of advanced chronic liver disease. McCormack et al. coined, for the first time in 1985, the term “portal hypertensive gastropathy” for congestion-related gastric mucosal alterations with no inflammation seen during endoscopy [5, 6]. Although its mechanism has yet to be exactly known, it was shown to be histologically characterized by the dilatation of mucosal and submucosal gastric vessels [3, 4]. Gastric mucosal response to mucosal damage alters in the stomach, and a complex of structural alterations is seen in all the components of the gastric wall in PHG. It has been argued that in such a case, mucosal hypoxia, impaired mucosal defense, impaired recovery skills, and alterations in epithelial cell integrity occur and the gastric mucosa becomes more vulnerable against all these alterations, leading to PHG development [5, 7, 8].

Gastric polyps are lesions that originate from gastric epithelia and grow towards the lumen. They are usually asymptomatic and randomly identified during upper GI endoscopy (EGD) [1, 2]. Although portal hypertensive gastropathy in liver cirrhosis is a well-defined finding in EGD in the literature, portal hypertension-associated gastric polyps have not yet been entirely defined. Their etiology is not known either.

In their study, Livovsky et al. carried out gastroscopic evaluation of 16 patients and concluded that those with advanced chronic liver disease had more polyps than the normal population. The authors found no dysplasia and malignancy finding in any of the polyps [5].

Gastroscopy results reveal that esophageal varices, fundal varices, and portal hypertensive gastropathy are well-defined endoscopic findings in cirrhotic patients. Yet, studies on portal hypertension-associated polyps are rather limited in the literature with some recent case reports or studies conducted with small series [2, 9–13]. In their study, Kara D. et al. evaluated the cases of 407 cirrhotic patients in this regard and found that 10% of the patients had portal hypertensive gastric polyps while reporting that most of these were hyperplastic polyps according to histopathological analyses. The authors determined that only 2.8% of these were adenoma. When the authors followed up all the polyps as ascertained by gastroscopy for 44.6 ± 14.7 months, they found no malignant transformation in any of the polyps. As was the case in our study, most of the polyps here, too, were located in the gastric antrum and prepyloric areas. The authors also reported in this study that the rate of gastric polyps was 10% while the rate of duodenal polyps was 8% with higher rates than those of the normal population when they assessed gastroduodenal polyp frequency [9]. Lemmers et al., on the other hand, reported a rather low rate of portal hypertension-associated polypoid lesions found in the stomach and small intestines in 14 patients (0.9%) in their 1538-patient study [2].

The prevalence of portal hypertensive polyps varies between 1 and 3% [11, 12, 14]. Amarapurkar et al. found in their study covering 3811 upper GI endoscopies that the prevalence of portal hypertensive polyps was 3.2%, similar to that of the normal population [4]. The prevalence of gastroduodenal polyps in the normal population varies between 0.5% and 6.35% [9, 15].

The results of our study, however, revealed a high rate of gastric polypoid lesions in cirrhotic patients in comparison to former studies. In our study, 67 (33.5%) out of 200 cirrhotic patients had polypoid lesions, while 20 (10.0%) patients in the normal healthy group had gastric polypoid lesions. When the two groups were compared with regard to polypoid lesions, it was seen that the rate of polypoid lesions was statistically higher in the advanced chronic liver disease group (*p* value < 0.001).

The reason why the number of polyps is so high in our study group might be the patients' advanced Child Pugh stages. All of them had esophageal varices, and most of them had ascites.

Pathological analyses of portal hypertensive polyps in studies revealed foveolar hyperplasia, edema in lamina propria, and lamina propria ectatic capillaries. None had dysplasia and malignancy [5, 9, 16].

Some authors declared that portal hypertensive polyps with diameters more than 10 mm have increased malignancy risks [11]. In our study cohort, the diameters of portal hypertensive polyps differed between 2 and 6 mm. Pathologists analyzed the portal hypertensive polyps in our study and concluded that none had any dysplasia and malignancy finding as well.

Amarapurkar et al. conducted immunohistochemical analyses of portal hypertensive polyps, and when they compared them to nonportal hypertensive polyps and normal gastric mucosa, they found that there was an increase in vessel density in the portal hypertensive mucosa and portal hypertensive polyps with a quite enlarged vessel diameter of >50 *μ*m [4].

Portal hypertensive polyps are histologically similar to hyperplastic polyps [9, 11], and they cannot be distinguished from the latter macroscopically either, but studies on the subject found that these portal hypertensive polyps had more small ulcerations on them when macroscopically evaluated [16, 17]. The results of our study also revealed that such polyps mostly had small ulcerations on them.

Lam et al. reported in their study that the mean diameter of portal hypertensive polyps was 6.7 mm (between 2 and 18 mm) and their histopathological results pointed to mucosal hyperplasia, extensive vascular proliferation, and granulation tissue formation [11]. The diameters of portal hypertensive polyps in our study varied between 2 and 6 mm, and they were generally large in number, side-by-side and adjacent having usually been localized in the gastric antrum and corpus.

When the patient and healthy groups' polyps were compared as to Helicobacter pylori (H. pylori) presence, it was observed that H. pylori was seen in 63 (31.5%) persons in the patient group while it was seen in 66 (33%) in the healthy group. In spite of the fact that H. pylori was slightly higher in the patient group, there was no statistically significant difference between the two groups (*p* < 0.09). In another study, however, the H. pylori rate in hyperplastic polyps was found to be 18% while no H. pylori could be detected in portal hypertension-associated polyps [11].

In another study, researchers found H. pylori in both groups and reported that the H. pylori rate went down in patients with advanced chronic liver disease mostly due to antibiotic administration while the growth of polyps in these groups was correlated with age [5].

The results of our study revealed that the rate of PHP was higher in patients with esophageal variceal endoscopic band ligation (EVL) than those without EVL and they statistically risked PHP formation 2.891 times more.

Similarly, Kara et al. found that those who previously received esophageal variceal EVL had more PHP than those who did not in line with the results of our study. Previous band ligation (EVL) appears to be a strong risk factor in the development of these polyps. The reason may be related to the fact that esophageal variceal band ligation leads to an increase in portosystemic shunt formations including the gastric wall. This hypothesis is also consistent with the histological finding of proliferative ectatic vessels in the gastric mucosa and strongly supports the hypothesis that elevated portal blood flow on the gastric mucosa distinctively stimulates proliferation [9].

The pathogenic mechanism of PHP has not been entirely uncovered yet, but some researchers have argued that the congestion brought about by elevated portal pressure might have been playing an important role in inducing proliferation and angiogenesis [2, 9, 17]. Moreover, it is also possible that mucosal damage, including the vascular structure as well, may be involved in the pathogenesis of portal hypertension-related polyps rather than the superficial inflammation of the mucosa [11]. In such a case, some observations suggest that these polyps might respond to the treatment of portal hypertension [2, 9, 17].

No clear pathological diagnostic criterion for portal hypertensive polyps has been identified yet [9, 12]. Nevertheless, foveolar epithelial hyperplasia and proliferation in ectatic capillary vessels at lamina propria, which are typical histological features of portal hypertensive polyps, are recognized as indicators of portal hypertensive polyps. And they can be differentiated from inflammatory polyps through such histopathological features [4, 9, 15, 16].

These PHP lesions are typically located at the gastric antrum, mostly multiply, and demonstrate typical microscopic findings. The fact that they are seen more often in advanced cirrhosis cases, notably in those with previous EVL procedures, reveals that portal hypertension plays an important role in the pathogenesis of PHP. Currently, there is no evidence as to the possibility that such polyps have a malignant potential. These portal hypertensive polyps risk hemorrhaging.

In conclusion, portal hypertensive polyps can be seen in the stomach and small intestines in patients with advanced liver disease who develop portal hypertension. Although its pathogenesis is not exactly known, the most widely accepted argument is that it forms due to congestion-related vascular dilatation. Studies in the literature have not reported any cases of dysplasia and malignant transformation. PHPs are seen more in patients with EVL, and they risk hemorrhaging but the risk decreases with the treatment of portal hypertension [2]. The portal hypertension of these polyps, referred to as “portal hypertensive polyps” in the literature, can be accepted as a gastroscopic finding of portal hypertension. Large, multicentered, prospective studies are needed to confirm these hypotheses.

## Figures and Tables

**Table 1:** The etiological factors identified in the cirrhotic group.

	Frequency (*n*)	Percent (%)	Valid percent	Cumulative percent
HBV	63	31.5	31.5	73.3
Cryptogenic	35x	17.5	17.5	86.8
NASH	33	16.5	16.5	95.0
Alcohol	30	15	15	57.5
PBS	18	9	9	99.5
HCV	11	5.5	5.5	76.8
Cardiac	3	1.5	1.5	78.0
HDV	2	1	1	77.3
Wilson's disease	2	1	1	100.0
HBV+HDV	1	0.5	0.5	73.5
HBV+NASH	1	0.5	0.5	73.8
HBV+alcohol	1	0.5	0.5	74.0
Total	200	100.0	100.0	

**Table:** (a) Age

Diagnosis	Mean (age)	*N* (number)	Std. deviation	Median	Minimum	Maximum
With PH	62.1300	200	12.29426	63.0000	27.00	90.00
Control	52.3550	200	16.01598	52.0000	18.00	87.00
Total	57.2425	400	15.07537	59.0000	18.00	90.00

**Table:** (b) Sex, male/female rates

	Diagnosis		Total
	Cirrhotic	Control	
Sex			
M	118	89	207
F	82	111	193
Total	200	200	400

**Table 3:** The number of polypoid formations in the cirrhotic and control groups.

	Diagnosis		*p*
Total polypoid lesions, *n* (%)	Cirrhosis *n* (%)	Dyspepsia *n* (%)	
Present, 87 (21.8%)	67 (33.5)^a^	20 (10.0)^b^	<0.001
Absent, 313 (78.2%)	133 (66.5)	180 (90.0)	

**Table 4:** H. pylori presence in the groups (*n*).

	Diagnosis		Total	*p*
	Cirrhosis	Dyspepsia		
H. pylori				
Present	63	66	129	*p* < 0.09
Absent	137	134	271	
Total	200	200	400	

## Data Availability

The data used to support the findings of this study are available from the corresponding author upon request. The data consists of the results and statistical analysis.
